# A complicated case of pachydermoperiostosis with spondyloarthritides: a case report

**DOI:** 10.1186/1752-1947-7-268

**Published:** 2013-12-13

**Authors:** Qing Zhang, Min Shen, Bo Yang, Keyi Yu

**Affiliations:** 1 Department of Internal Medicine, Peking Union Medical College Hospital, Chinese Academy of Medical Science & Peking Union Medical College, Beijing 100730, China; 2 Department of Rheumatology, Peking Union Medical College Hospital, Chinese Academy of Medical Science & Peking Union Medical College, Key Laboratory of Rheumatology and Clinical Immunology, Ministry of Education, Beijing 100730, China; 3 Department of Orthopedics, Peking Union Medical College Hospital, Chinese Academy of Medical Science & Peking Union Medical College, Beijing 100730, China

**Keywords:** Bisphosphonate, Pachydermoperiostosis, Spondyloarthritides, Synovectomy, Zoledronic acid

## Abstract

**Introduction:**

Pachydermoperiostosis is a rare, hereditary disease commonly presenting with digital clubbing, pachydermia and periosteal hypertrophy. Therapeutic options for pachydermoperiostosis are few because of the unknown pathogenesis. Here, we report the complicated case of a patient with pachydermoperiostosis combined with spondyloarthritides, who was refractory to steroids and tumor necrosis factor alpha antagonists. We treated this patient with zoledronic acid and performed an arthroscopic synovectomy, with a satisfactory outcome. To the best of our knowledge, this is the first report in English on the combination of zoledronic acid administration and synovectomy for the treatment of a patient with pachydermoperiostosis and spondyloarthritides.

**Case presentation:**

An 18-year-old Han Chinese man was diagnosed with pachydermoperiostosis in the presence of digital clubbing, periostitis and swollen limbs. Combined spondyloarthritides was also considered based on his lower back pain, lower limbs synovitis, bilateral sacroiliac sclerosis and a positive test for human leukocyte antigen B27, as well as immunoglobulin A nephropathy. He was refractory to steroids and tumor necrosis factor alpha antagonists, but treated with intravenous zoledronic acid and an arthroscopic synovectomy, with a satisfactory outcome.

**Conclusion:**

This is a rare, complicated case of pachydermoperiostosis with spondyloarthritides. Combination therapy of zoledronic acid administration with synovectomy is a novel, convenient and effective option for patients with pachydermoperiostosis with remarkable synovitis.

## Introduction

Pachydermoperiostosis is a rare, hereditary disease commonly presenting with digital clubbing, pachydermia and periosteal hypertrophy [1]. In the past couple of decades, there have been several reports and some research aimed at finding more effective treatment methods for this disease. We report the complicated case of a patient with pachydermoperiostosis and spondyloarthritides who was refractory to steroids and tumor necrosis factor alpha (TNF-α) antagonists, treated by administration of zoledronic acid and an arthroscopic synovectomy with a satisfactory outcome. To the best of our knowledge, this is the first report in English of a combination of zoledronic acid administration and a synovectomy for pachydermoperiostosis therapy.

## Case presentation

An 18-year-old Han Chinese man presented to our hospital with a two-year history of pain and swelling in multiple joints, with facial changes of one year duration.

**Figure 1:**
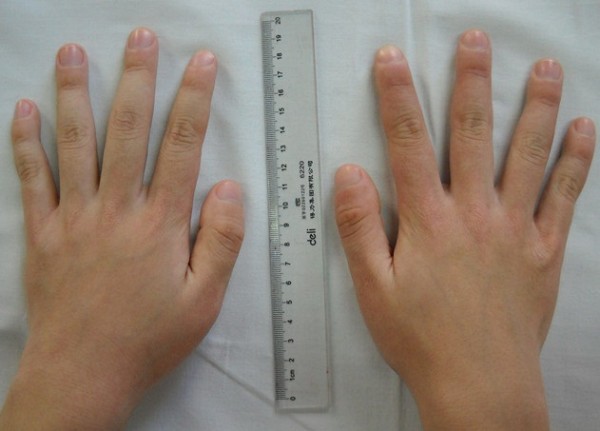
Enlargement of the hands with digital clubbing.

In the winter of 2008 he began to experience joint pain involving his knees, ankles, hips and the small joints of his hands with obvious swollen knees and ankles. Gradually, he developed difficulty squatting and clenching his fists, as well as lower back pain with morning stiffness. He denied fever, weight loss, eye diseases or inflammatory bowel diseases. He was diagnosed with spondyloarthritides and treated with steroids, salicylazosulfapyridine, loxoprofen sodium and infliximab, but without positive effects. In April 2009, minimal proteinuria and hematuria were found. A kidney biopsy showed immunoglobulin A (IgA) nephropathy with a Lee’s classification of Grade I. He further developed overt facial acne, an oily face with excessive desquamation, furrowing of his facial, scalpel and plantar skin, hyperhidrosis and enlargement of his hands and feet with digital clubbing (Figure 1).

**Figure 2:**
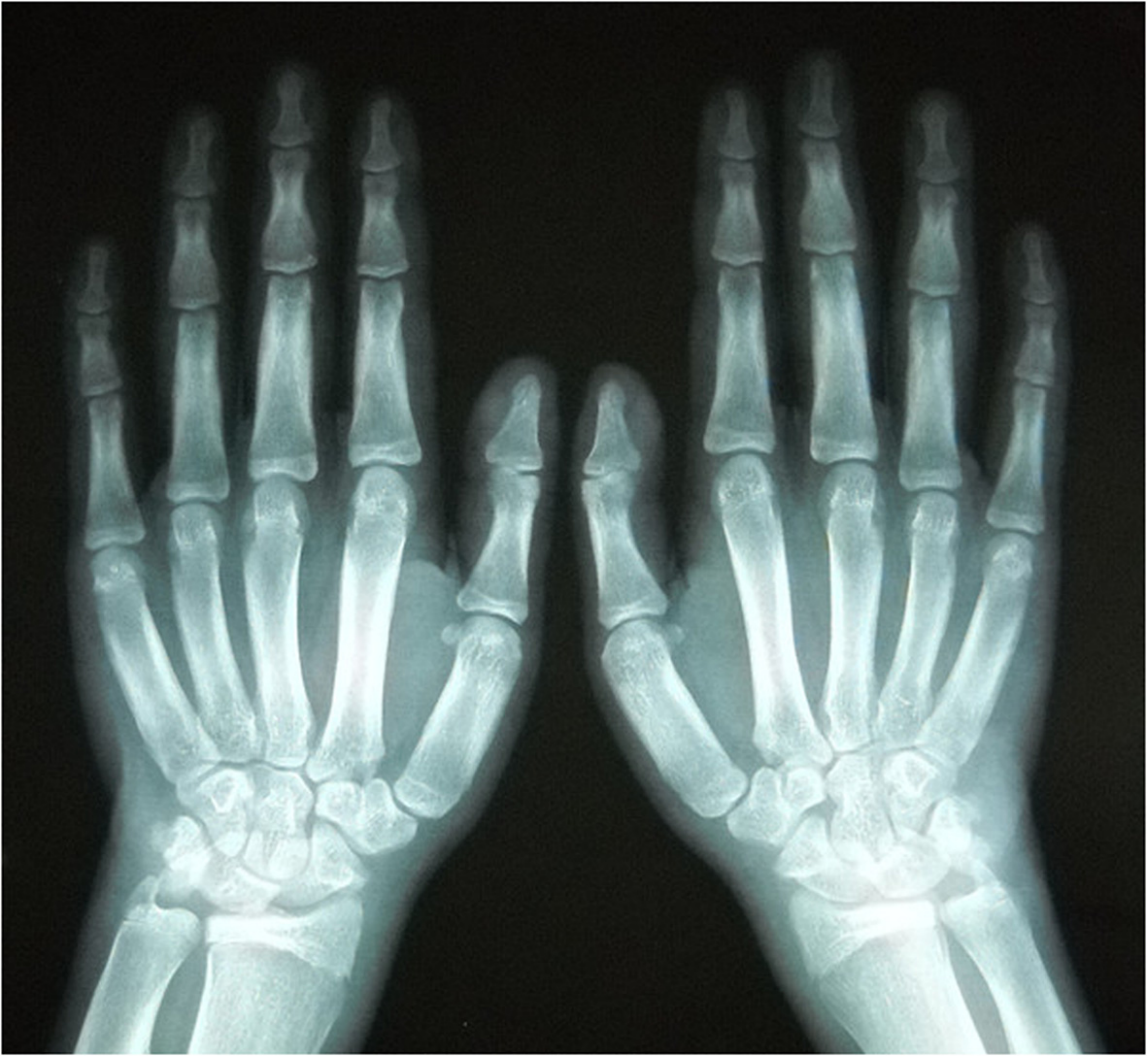
X-ray images of the hands disclosed widening of the epiphysis.

A physical examination found no limitation of his chest expansion or lumbar spine mobility. A serum investigation showed a two-fold elevation of erythrocyte sedimentation rate (ESR) and a 10-fold elevation of C-reactive protein (CRP); he was negative for rheumatoid factor and positive for human leukocyte antigen B27. An ultrasound of his knees indicated synovitis. Joint effusion showed inflammation with a negative culture result. Radiographs of his hands and feet demonstrated marked clubbing with mushrooming of the tufts (Figure 2). Periostosis was detected in his tibiae and fibulae, limited to the diaphysis, with a monolayer configuration. A sacroiliac computed tomography scan revealed bilateral blur and sclerosis with widened joint gaps.

After admission to our hospital, our patient was diagnosed with pachydermoperiostosis complicated by spondyloarthritides, and given prednisone 50mg/day, methotrexate 15mg/week and salicylazosulfapyridine 2g/day, as well as calcium and alendronic acid 70mg/week. This combined therapy yielded a poor response. He was then administered intravenous zoledronic acid and we performed a synovectomy on both knee joints, with prednisone tapered rapidly over the following two weeks. His symptoms improved remarkably, with an assessment of pain intensity recorded on a 10cm visual analogue scale decreasing from 8 to 1. His ESR and levels of CRP also decreased to normal. Our patient recovered and remains well six months after discharge.

## Discussion

Pachydermoperiostosis is one of the conditions that makes up the primary hypertrophic osteoarthropathy (HOA) group, and is a rare hereditary disease occurring predominantly in men. Clinical symptoms include the ‘HOA triad’ of digital clubbing, periostitis and swollen limbs. Periostosis shown in radiographs is also used in the diagnosis of HOA. Elevated ESR and CRP with synovitis might be found in patients with pachydermoperiostosis, yet it is fairly rare to note a two-fold elevation of ESR and a 10-fold elevation of CRP. Our patient had lower back pain with morning stiffness, lower limb joints synovitis and bilateral sacroiliac sclerosis, plus was positive for human leukocyte antigen B27. IgA nephropathy is common in patients with spondyloarthritides, but there is no such association in pachydermoperiostosis. A diagnosis of spondyloarthritides based on IgA nephropathy is thus considered reasonable. The failure to respond to TNF antagonists might be because this sclerosing bone-forming phenotype of spondyloarthropathy is less responsive to TNF antagonists than other more inflammatory phenotypes. Moreover, a good response to zoledronic acid in patients with spondyloarthritides has previously been described [2]. Considering all these factors, our patient was finally diagnosed with pachydermoperiostosis complicated with spondyloarthritides, plus IgA nephropathy. Only one case of pachydermoperiostosis associated with spondyloarthritides has previously been reported [3], which may have been a coincidence, but an association between these two diseases could cause diagnostic problems.

There are several possible etiological factors for pachydermoperiostosis, such as genetic aberration, fibroblast abnormality, peripheral blood flow alteration, higher osteoblastic activity and excess fibroblast proliferation. But therapeutic options for pachydermoperiostosis are few because of the unknown pathogenesis. Conventional drugs like non-steroidal anti-inflammatory drugs, pain-killers and colchicine [4] are usually the first-line drugs. Plastic surgery can be an option for patients with remarkable facial changes. We considered that reducing the inflammatory component and the osteoclastic or osteoblastic activity might be an effective therapy. Nevertheless, steroids, disease-modifying anti-rheumatic drugs and infliximab all failed in our patient.

Although several case studies have reported that parenteral bisphosphonates can reduce pain and other symptoms related to HOA [4-6], alendronic acid was tried in our patient without response. In one study, pamidronate resulted in a significant reduction of pain in three patients with HOA. In other studies, zoledronic acid was shown to be effective [7-9]. We treated our patient with intravenous zoledronic acid and noted a rapid remission with no adverse events. Compared with other bisphosphonates, zoledronic acid is novel, effective and more convenient. Long-term follow-up should be recommended. The mechanism of action of bisphosphonates in HOA remains unclear but it is commonly accepted that they have both anti-resorptive and anti-inflammatory benefits [7].

There were also several reports of patients with pachydermoperiostosis treated with synovectomy [5]. With regard to an associated obvious synovitis and refractory elevated ESR and CRP, a synovectomy was carried out in our patient. Post-surgical levels of ESR and CRP decreased to normal ranges. It appears that synovectomy is effective in the obvious inflammatory reaction in pachydermoperiostosis, both for local and systemic inflammation.

## Conclusion

This is a complicated case of a patient with pachydermoperiostosis with spondyloarthritides and IgA nephropathy. We highlight the difficulties encountered in the clinical practice of diagnosis and treatment. A combination therapy of zoledronic acid administration and synovectomy is a novel, convenient and effective option for patients with pachydermoperiostosis with remarkable synovitis.

## Consent

Written informed consent was obtained from the patient for publication of this case report and accompanying images. A copy of the written consent is available for review by the Editor-in-Chief of this journal.

## Abbreviations

CRP: C-reactive protein; ESR: Erythrocyte sedimentation rate; HLA: Human leukocyte antigen; HOA: Hypertrophic osteoarthropathy; IgA: Immunoglobulin A; TNF: Tumor necrosis factor

## Competing interests

The authors declare that they have no competing interests.

## Authors’ contributions

QZ was involved in the literature review, manuscript preparation and editing. MS was involved in the conception of the report, manuscript critique, review and submission, and was a major contributor in writing the manuscript. BY and KY performed the arthroscopic synovectomy and were involved in the manuscript critique and review. All authors read and approved the final manuscript.
